# Declarative Memory Predicts Phonological Processing Abilities in Adulthood

**DOI:** 10.3389/fpsyg.2021.658402

**Published:** 2021-05-25

**Authors:** Dana T. Arthur, Michael T. Ullman, F. Sayako Earle

**Affiliations:** ^1^Department of Communication Disorders, State University of New York–New Paltz, New Paltz, NY, United States; ^2^Department of Neuroscience, Georgetown University, Washington, DC, United States; ^3^Department of Communication Sciences and Disorders, University of Delaware, Newark, DE, United States

**Keywords:** procedural memory, declarative memory, learning, phonological processing, phonological awareness, nonword repetition

## Abstract

Individual differences in phonological processing abilities have often been attributed to perceptual factors, rather than to factors relating to learning and memory. Here, we consider the contribution of individual differences in declarative and procedural memory to phonological processing performance in adulthood. We examined the phonological processing, declarative memory, and procedural memory abilities of 79 native English-speaking young adults with typical language and reading abilities. Declarative memory was assessed with a recognition memory task of real and made-up objects. Procedural memory was assessed with a serial reaction time task. For both tasks, learning was assessed shortly after encoding, and again after a 12-h, overnight delay. We regressed phonological processing ability with memory performance on both days. We found that declarative memory, but not procedural memory, was highly predictive of phonological processing abilities. Specifically, declarative memory scores obtained shortly after learning were associated with non-word repetition performance, whereas declarative memory scores obtained after the overnight delay were associated with phonological awareness. Procedural memory was not associated with either of the phonological processing measures. We discuss these findings in the context of adult participants with mature phonological systems. We examine possible implications for the relationship between declarative memory and phonological processing in adulthood.

## Introduction

The purpose of this research was to investigate potential roles of learning and memory systems in phonological processing. Phonological processing supports spoken language processing throughout the lifespan, and is considered a foundational skill in the development of literacy in alphabetic writing systems (Pennington et al., 1990; Adams, 1996). As such, phonological processing is implicated in various idiopathic developmental disorders of language and reading (Nation and Hulme, 1997; Scarborough, 1998; Ramus, 2001; Archibald and Gathercole, 2006; Estes et al., 2007). Thus, the skills and capacities that account for individual differences in phonological processing have been the subject of extensive research. These previous investigations have largely focused on perceptual influences (see below). By comparison, there has been little inquiry into how individual differences in learning and memory play a role; this study begins to fill this gap.

Phonological processing refers to the use of speech-sound information in the processing of oral and written language (Wagner and Torgesen, 1987; Brady and Shankweiler, 1991). It encompasses various capacities, including phonological awareness (PA), phonological working memory, and phonological retrieval (Wagner and Torgesen, 1987). PA is the ability to consciously manipulate sub-lexical phonological segments. PA skill development is predicated by the awareness of phonological elements and patterns within one’s language. PA is typically measured by performance on tasks such as phoneme blending (sounds are presented one phoneme at a time, and the examinee must put them together to make a whole word) or segmentation (examinees are given a word, and asked to remove pieces of the word to make a different word). Phonological working memory refers to the short-term storage of phonological information (within a sound-based representational system), which is then used in the service of additional processing tasks (Baddeley, 1996). Thus, PA and phonological working memory are similar capacities that require the retrieval and manipulation of phonological segments. Non-word repetition (NWR) is a task that is often used to index phonological working memory. Nevertheless, NWR is generally not considered a pure index of phonological working memory, but instead incorporates multiple additional skills and knowledge (e.g., vocabulary, phonology, and composition; Dillon et al., 2004; Gupta and Tisdale, 2009; Krishnan et al., 2013; Dye et al., 2016). We note that NWR is nevertheless a worthwhile measure of phonological working memory function within the framework of phonological processing precisely *because* this task relies on the cooperation between working memory and knowledge about phonological segments.

Phonological processing is used clinically as an index of language function. Given the various capacities recruited for phonological processing, it is unsurprising that scores obtained on phonological processing tasks predict speech and language skills across typical and disordered populations, in children and in adults (Casserly and Pisoni, 2013; Adlof and Patten, 2017; Del Tufo and Earle, 2020; Earle and Del Tufo, 2021). Indeed, difficulties in phonological processing are often considered a hallmark of language and reading disorders. Moreover, differences in symptoms between various disorders are often considered the result of differences in the nature of the phonological processing difficulties (Ramus et al., 2013; Ehrhorn et al., 2020). Phonological processing has long been considered an early intervention point for those at risk of developing language and reading disorders (Vandervelden and Siegel, 1997; Torgesen et al., 1999a). Overall, there are thus both theoretical and clinical stakes in understanding the component skills and abilities that account for individual differences in phonological processing.

The existing literature on the factors that may account for individual differences in phonological processing has largely focused on perceptual factors. One body of work has attributed differences in phonological processing abilities to differences in auditory perception involved in the analysis of speech acoustics, particularly in the work of Tallal et al. (1993) and Stark et al. (1988). Alternatively, it has been proposed that differences in speech perception, rather than low-level auditory abilities, account for variability in phonological processing (Joanisse and Seidenberg, 2003). These proposals share the perspective that variability in phonological skills reflect factors that affect the online processing of speech. However, given that phonological processing is not a monolithic skill, there are likely to be other loci of individual differences beyond perceptual abilities that inform differences in phonological processing.

Of interest here, the role of pre-existing phonological knowledge, and how that knowledge becomes established, also warrants consideration. We focus on declarative and procedural memory, two brain systems that are important for various aspects of language learning and representation (Ullman, 2004; Ullman et al., 2020). The declarative/procedural model of language (Ullman, 2004; Ullman et al., 2020) is built on a neural dissociation between the two systems (Doyon et al., 2009; Ashby et al., 2010; Eichenbaum, 2012; Squire and Wixted, 2011). Declarative memory, which is defined as the learning and memory that rely on the medial temporal lobe and associated circuitry, underlies (at least) episodic and semantic information. Procedural memory, which is defined as the learning and memory that rely on the basal ganglia and associated circuitry, underlies the learning and automatizing of motor and cognitive skills and habits. Both systems have been implicated in learning various aspects of language (for reviews, see Ullman, 2004; Ullman et al., 2020). For example, evidence suggests that lexical knowledge is supported primarily by the declarative memory system. The acquisition and use of grammatical regularities, possibly including in phonology, are posited to rely on both memory systems, with the relative dependence on one versus the other system a function of various factors (Ullman, 2004; Ullman et al., 2020). Procedural memory is specifically hypothesized to subserve the learning and processing of grammatical knowledge that involves the real-time prediction of downstream elements (Ullman et al., 2020). In contrast, declarative memory is thought to underlie chunk-based schematic knowledge about grammatical constructions, based on experience with specific forms that exemplify that construction (Ullman et al., 2020).

This framework provides various predictions regarding the conditions under which phonological processing may rely on either declarative or procedural memory, although the extant literature favors the primacy of procedural memory in this capacity. For example, phonological processing performance that relies on the computation of probable, upcoming segments based on knowledge about phonotactic regularities in one’s language might be expected to rely on procedural memory. In addition, speech-sound category knowledge that emerges through the implicit learning of regularities in acoustic-phonetic features (via linguistic exposure) may be largely subsumed by the procedural memory system (Ullman et al., 2020). For these reasons, weaknesses in phonology in language and reading-impaired populations have been attributed to a proposed procedural memory deficit (Ullman and Pierpont, 2005; Lum et al., 2013; McGregor et al., 2013; Ullman et al., 2020). While this assumption may be valid concerning these populations, it does not necessarily follow that differences in phonological processing in general are wholly attributable to individual differences in procedural memory, particularly in adulthood.

Specifically, declarative memory contributes to both segmental (Chandrasekaran et al., 2014) and lexical (Ullman, 2004; Ullman et al., 2020) representations that comprise one’s phonological inventory. In speech-sound learning, adults appear to be more likely to utilize strategies that rely on declarative, over procedural, memory (Maddox et al., 2013). For lexical forms, there is ample evidence to support the role of declarative memory in learning sound sequences in single chunks (Ullman, 2004; Ullman et al., 2020). This includes evidence that suggests that novel word forms are integrated with the preexisting lexicon overnight (Dumay and Gaskell, 2007), consistent with offline consolidation of hippocampal memory (Dudai, 2004; Diekelmann and Born, 2010).

Lexical knowledge is considered a particularly important predictor of phonological processing ability, especially when measured by NWR. In investigations of the subskills supporting NWR performance, evidence has been found that vocabulary knowledge influences performance on NWR tasks, despite the non-lexical nature of task items (Metsala, 1999; Edwards et al., 2004). Metsala and Walley (1998) posit that this relationship illustrates the role of increased vocabulary skill in specifying phonological representations (lexical restructuring). Representations of lexical forms provide schematic exemplars of phonotactic construction that further contribute to knowledge about phonological rules. This may be particularly true in individuals with a mature vocabulary (e.g., in adults), who need not rely on probabilistic computations to arrive at phonological rules, but can abstract this information from first-hand experience with the language. Gupta and Tisdale (2009) contributed to the investigation of vocabulary, phonological short term memory, and NWR performance through the development of a computational model which operationalized their interaction. Their findings suggest that phonological working memory supports both NWR and vocabulary learning, while vocabulary size supports NWR performance. Finally, whereas phonotactic rules might be expected to have been largely learned in procedural memory over the course of childhood (like other aspects of grammar; Hamrick et al., 2018), lexical knowledge is likely continually updated during one’s lifetime, given that word learning does not stop in one’s early years. Thus, our declarative learning abilities during adulthood might continue to be relevant for lexical knowledge and so may influence phonological processing in adulthood, while adult procedural learning abilities may contribute less at this later stage of life. Together, the evidence thus supports the idea that declarative memory may have a larger role in at least some aspects of phonological performance than has been previously assumed, particularly in adulthood.

There is indeed some empirical evidence that learning and memory play a role in phonological processing, and that differences in some phonological processing skills may be explained by differences in learning and memory, including in memory consolidation. Memory consolidation refers to the different stages of memory processing that act upon a trace following exposure, and which may occur as a function of time and/or as a function of sleep (Dudai, 2004). In a recent investigation of non-native phonological-contrast learning, Earle and Arthur (2017) found that phonological processing skills in the native language of participants were not associated with their ability to learn non-native sound contrasts immediately after training in those contrasts. However, an association emerged between phonological processing and perceptual performance on the trained non-native contrast following a period of sleep. Specifically, following sleep, NWR scores predicted performance on the perceptual identification of the non-native contrast, while performance on a PA task predicted non-native sound discrimination. The emergence of associations between these tasks after sleep suggests that phonological processing performance is informed by one’s ability to access long-term information (acoustic-phonetic, in this particular case). Although it is clear that memory processes likely play a role, we are less certain about the particular types of memory that are involved. Specifically, we do not know if our prior observations with respect to a delayed association between phonological processing and speech-sound information reflect declarative memory, procedural memory, or both. Nevertheless, we note that enhanced performance following an overnight delay is consistent with offline, or sleep-mediated, consolidation often observed for declarative memory (see Diekelmann and Born, 2010, for review). In contrast, sleep has been argued not to play a critical role in the consolidation of procedural memory (Nemeth et al., 2010; c.f. Stickgold, 2005). Thus, the delayed association between phonological processing and perception of a non-native contrast observed in Earle and Arthur (2017) may reflect aspects of the speech-sound representation encoded in declarative memory.

To our knowledge, there has not been a direct empirical examination in adulthood of associations between learning in the two memory systems and performance on the phonological processing tasks described above. Therefore, the purpose of the current paper is to explore associations between procedural and declarative memory on the one hand, and performance on phonological processing tasks on the other. We note that declarative and procedural memory are not specific to any sensory modality, but are rather considered general-purpose learning systems across modalities and domains. Thus, empirical tests of this framework often examine the relationship between language functions and learning and memory abilities with non-auditory and non-verbal tasks (Clark et al., 2014; Hamrick et al., 2018; Earle et al., 2020). Indeed, the use of such tasks is critical to avoid obtaining false positive associations between declarative or procedural learning measures and language measures due to shared linguistic involvement (rather than a shared involvement of the memory systems) (Hamrick et al., 2018). Thus, to address our research objective, analyses were performed on a dataset collected previously that included performance on PA and NWR tasks, as well as non-verbal tasks of declarative and procedural memory assessed both shortly after learning and after a period of overnight delay.

We hypothesized that at least declarative and perhaps procedural memory might predict phonological processing performance. Further, any links with DM were expected to be more likely with NWR than with PA. Because of our prior observations that relationships emerged between speech-sound memory and phonological processing performance only after a delay (Earle and Arthur, 2017), we reasoned that one or both measures of phonological processing might show an association with a delayed measure of declarative memory, perhaps in addition to a measure taken shortly after learning. In contrast, as procedural memory may not show overnight enhancements (Nemeth et al., 2010), we expected to find a stable relationship between phonological processing and procedural memory across time.

## Materials and Methods

We present here the results of secondary analyses conducted on a dataset previously presented in Earle and Ullman (2021), with the purpose of addressing the current research questions. Scores on the tests of phonological processing featured as the outcome measure here have also been included previously in larger datasets on the skill profiles of college students (Del Tufo and Earle, 2020; Earle and Del Tufo, 2021).

### Participants

Data presented in the present paper were collected at the University of Connecticut (UConn) and the University of Delaware (UD). Participants at UConn were primarily recruited from the Psychology department participant pool and participated in exchange for course credit. Participants at UD were recruited through approved flyers and social media posts from within the University community. Participants at UD were given the option of receiving either course credit or compensation in gift cards at a rate of $10/h.

Criteria for study inclusion at both sites were as follows: participants must be native speakers of English (age 18–24) with no history of vision or hearing impairment, cognitive impairment, neurological injury, mood or attention disorders, or socio-emotional disorders. While the parent studies recruited across a wide range of language and reading ability, we include in the present paper only the subset of our sample without a history of disordered reading, speech, or language. This resulted in a sample of 79 participants across both sites (32 UConn, 47 UD), with a mean age of 20.48 years (2.43 standard deviation), and a male:female ratio of 26:53. The UConn sample was slightly younger on average (mean 18.94 years, 0.58 standard deviation) than the UD sample (21.54 years, 2.63 standard deviation). This may have been attributable to our recruitment procedures, in that the UConn participant pool was comprised mostly of first-year students.

### Procedures

All participants provided informed consent prior to participation, under procedures approved under separate protocols by the Institutional Review Boards at the respective sites. We note that the current analyses presented in this paper fall within the scope of the respective studies as described by the informed consent documents.

All participants first completed a 2-h test administration session, one-on-one with the experimenter in a small, quiet testing room. During this initial session, participants completed a battery of language, reading, and cognitive assessments. Participants were confirmed to meet the inclusionary criteria specified above through performance on these assessments. Specifically, participants whose data are included in the present study obtained scores at or above 1 standard deviation below the mean on tests of non-verbal cognition and word-level reading. Non-verbal cognition was measured via the Block Design and Matrix Reasoning subtests of the Wechsler Abbreviated Scale of Intelligence (Wechsler, 1999) at UCONN, and the Wechsler Abbreviated Scale of Intelligence-II (Wechsler, 2011) at UD. Word-level reading was assessed using the Word Attack and Word Identification subtests of the Woodcock Reading Mastery Test, Third Edition (WRMT-III; Woodcock, 2011) at both sites, and the Test of Word Reading Efficiency (Torgesen et al., 1999b) at UCONN and the Test of Word Reading Efficiency-2 (TOWRE-2; Torgesen et al., 2012) at UD. In addition, participants needed to be identified as having typical language by the screening method described by Fidler et al. (2011, 2013). Broadly described, this involves entering the raw scores obtained on a modified token task and a 15-word spelling test into a regression equation derived from a discriminant analysis performed on a large dataset. The resultant value indicates the presence/absence of language disorder in adults at 80% sensitivity and 87% specificity (Fidler et al., 2011, 2013).

During this test session, participants also completed assessments of phonological processing. Phonological processing was assessed using the Elision, Blending, and NWR subtests of the Comprehensive Test of Phonological Processing (CTOPP; Wagner et al., 1999) at UConn, and CTOPP-2 (Wagner et al., 2013) at UD. The Elision subtest requires participants to remove sub-lexical constituents of a real word to make another word (“Say popcorn. Now say popcorn without saying corn.”). The segments to be removed decreases in length over the course of the trials, thereby increasing the difficulty of the task. In the Blending subtest, participants are played a recorded sequence of segments and instructed to put them together to make a whole word (“What word do these sounds make?/m/…/ae/…/d/”). The number of segments to combine increases over the course of the trials. Performance on the Elision and Blending subtests are both considered measures of PA. In the NWR subtest, participants are played recorded pseudowords and instructed to repeat the word as clearly and as accurately as they can (“Listen to the made up word. Then say it exactly as you hear it./ttexteshɑsd/”). The pseudowords increase in length and complexity over the course of the trials. While a discussion of the remaining measures obtained during this session is beyond the scope of the current paper, a description of the full test battery, along with test scores obtained by the current sample, is reported in Earle and Ullman (see Table 1, S1, under “TD adults”; 2021). We note that the CTOPP and CTOPP-2 differ only in the lengths of these subtests. That is, the CTOPP-2 uses the same exact stimuli used in CTOPP for the Elision, Blending, and NWR subtests, however, the CTOPP-2 contains 10–14 additional items. A small number of the additional trials occur early in the revised subtests, but most of these items extend the ceiling of the CTOPP. Thus, there was the potential for the raw scores to substantially differ according to site. This was dealt with via data transformation, as described below. Following test administration, subtest scores were calculated from raw score sheets by two scorers, and discrepancies in scoring (<2%) were flagged and resolved by the last author.

**TABLE 1:** Describes the average and standard deviations of performance on outcome measures.

Experimental task performance by site				

		UConn	UD	Average
		*n* = 32	*n* = 47	
**Phonological processing performance (raw scores)**				
Elision		18.72 (0.89)	31.09 (1.51)	–
Blending		17.06 (2.11)	28.84 (2.67)	–
Non-word Repetition		14.31 (1.91)	20 (2.15)	–
**Serial reaction time performance (ms)**				
Day 1	Random	492.84 (62.23)	476.15 (81.33)	483.00 (75.85)
	Sequence	439.61 (69.88)	430.60 (92.53)	434.30 (83.60)
	Difference	53.23 (27.58)	45.54 (46.78)	48.70 (39.99)
Day 2	Random	439.07 (58.00)	417.86 (58.12)	426.29 (58.63)
	Sequence	400.65 (60.86)	383.79 (62.81)	390.49 (62.20)
	Difference	38.42 (23.13)	34.07 (29.27)	35.80 (26.92)
**Recognition memory performance (*d*’)**				
Day 1	Real	1.68 (1.23)	2.01 (0.91)	1.88 (1.05)
	Made up	1.81 (1.31)	0.56 (0.45)	1.07 (1.09)
	Average	1.75 (1.20)	1.28 (0.62)	1.47 (0.93)
Day 2	Real	2.14 (1.23)	2.35 (0.91)	2.27 (1.05)
	Made up	2.02 (1.42)	0.98 (0.52)	1.41 (1.11)
	Average	2.08 (1.29)	1.67 (0.62)	1.84 (0.96)

*Measures of phonological processing were obtained on the Elision, Blending, and Non-word Repetition subtests of the Comprehensive Test of Phonological Processing (CTOPP); Wagner et al. (1999) at University of Connecticut (UConn), and CTOPP-2 (Wagner et al., 2013) at University of Delaware (UD) For the serial reaction time task, the values expressed are the average reaction times of all accurate trials in the last random block (Random) and the last sequence block (Sequence) of each day. For recognition memory performance, the values are expressed in *d’* (Macmillan and Creelman, 2004) for recognition trials on real objects (Real) and made up (Made Up) objects.*

Following the initial testing session, participants were scheduled for a two-session experiment. Session 1 took place from 7:30 to 9 P.M., and Session 2 took place at 8 A.M.–9 A.M. on the following morning. During Session 1, participants completed the declarative memory task, followed by the procedural memory task. Participants were assessed in their performance on the memory tasks both shortly after learning during Session 1, and again during Session 2.

#### Declarative Memory: Recognition Memory Task

The recognition memory task was developed by the second author, and was adapted from a version previously used to assess declarative memory in both children and adults (Lukács et al., 2017; Earle et al., 2020; Reifegerste et al., 2021). Specifically, the task is designed to recruit the neuroanatomical structures of the declarative memory system, while reducing attentional and working memory demands on task performance (Hedenius et al., 2013; Lukács et al., 2017; Reifegerste et al., 2021). Importantly, we chose a recognition memory task in a visual modality in order to avoid the potential for associations between learning and phonological processing measures.

Visual stimuli for the recognition memory task included black-and-white drawings of real (64) and made-up (64) objects. The images of real objects were obtained from clipart galleries as well as from the line drawings of Snodgrass and Vanderwart (1980). These objects were carefully selected to match across sets utilized in the learning and test phases on word frequency, number of syllables, and number of phonemes. Images of made-up objects were selected for their low nameability and modified from stimuli used in previous studies (Eals and Silverman, 1994; Williams and Tarr, 1997; Cornelissen et al., 2004). Images of real and made-up objects were modified and retouched as necessary to achieve comparable size and composition.

During Session 1, participants completed the encoding phase of the recognition memory task, followed by the recognition phase approximately 10 min later. Participants were seated in front of a computer screen and instructed to place the index finger of each hand on marked keys (“s” and “l”) on the keyboard. Participants were given three practice trials, wherein an image appeared on the screen, and participants were asked to indicate, as quickly and as accurately as possible, whether the images represented real or made-up objects. After completing the practice trials, participants were given 64 trials of this task (32 real/32 made-up), each beginning with a 1-s fixation cross at the center of the screen. Each object was presented on the screen for exactly 500 ms, in order to ensure equal duration of exposure to each stimulus item. Trials ended at 500 ms if the response occurred during the stimulus presentation; or, if the response occurred after 500 ms, a fixation cross replaced the object until the participants indicated their response. Images were presented in a pre-set, pseudorandom order that avoided more than three consecutive trials of the same stimulus type (real/made-up).

During the subsequent recognition phase, participants were again asked to place an index finger from each hand on marked keys (“s” and “l”) on the keyboard. Participants first completed six practice trials, wherein an image appeared on the screen, and participants were asked to indicate, as quickly and as accurately as possible, if the image had been seen before during the encoding phase. Following the practice trials, participants completed 128 trials of this task (all 64 items previously seen/64 not previously seen), with the trial structure mirroring that of the encoding phase.

During Session 2, participants completed the retention phase of the experiment. The retention phase utilized an identical task structure as the recognition phase of Session 1. Another 128 trials were completed in the retention phase (all 64 items previously seen/64 not previously seen) with a new set of foils, that is, items that were not seen during the encoding task.

While no task provides a perfect measure of any given cognitive construct, a particular limitation regarding the nature of this declarative memory task is that performance may be aided in part by lexical knowledge for the real items. We note that one need not know what something is called to judge whether it’s real (as during the encoding phase), or whether something has been seen before (as during the recognition phase). This concern is somewhat mitigated by the inclusion of recognition of made-up items in the composite declarative memory score (see below), as recognition memory performance on made-up items is unlikely to rely on pre-existing lexical knowledge.

#### Procedural Memory: Serial Reaction Time Task

Procedural memory skills were assessed using a version of the serial reaction time task originated by Nissen and Bullemer (1987). Performance on this task has been found to rely on neuroanatomical structures underlying procedural memory (see Clark et al., 2014, for meta-analysis), and is therefore often used to index procedural memory function.

During Session 1, participants were seated in front of a computer in a quiet room, and instructed to put four fingers from their dominant hand on marked, consecutive keys on the computer keyboard. Four horizontally arranged boxes appeared on the screen. Participants were instructed to watch for the smiley face, and to hit the key corresponding to its location as quickly as possible when the smiley face appears. Participants first completed a warm-up block of 40 trials in which the stimulus appeared in a pseudorandom order. This was followed by four blocks of 80 trials in which the stimulus occurred in a fixed, 10-trial sequence of locations (i.e., the sequence was repeated eight times per block). Finally, participants completed an additional block of 80 pseudorandomized trials. During Session 2, participants completed the warm-up block of 40 pseudorandom trials, followed by one block of 80 sequence trials, and one block of 80 pseudorandom trials.

## Analyses and Results

Due to either time constraint during the initial testing session or equipment malfunction during the experiment Sessions, we are missing information about phonological processing from four participants, and from one participant each on procedural and declarative memory performance. Missing cases were estimated using the Multivariate Imputation by Chained Equations (package “mice”, van Buuren et al., 2015), in R (R Development Core Team, 2017; Version 3.4.1, 2017).

### Descriptive Results

See Table 1 for summary of performance measures across sites.

#### Phonological Processing

As described above, participants at UConn were administered the CTOPP (Wagner et al., 1999), and the CTOPP-2 (Wagner et al., 2013) at UD. Thus, the raw scores differ greatly by site (see Table 1). See “Data Transformations” below for a description of the scaling procedure applied prior to analyses for this reason.

#### Declarative Memory

We calculated *d’* scores as our index of declarative memory from accuracy (% trials correct) during the recognition and retention phases of the task. *D’* is defined as the difference between the z-scores of the hit and false alarm rates (Macmillan and Creelman, 2004; Hedenius et al., 2013; Earle et al., 2020). In the context of the recognition memory task, the hit rate corresponds to the percentage of familiar trials correctly identified as “seen before.” The false alarm rate corresponds to the percentage of unfamiliar trials incorrectly identified as “seen before.” This measure is often used as an index of perceptual sensitivity which accounts for response bias. As we could not be certain that the response bias would be uniform for real and made-up items, *d’* scores for item types were computed separately for real and made-up items, then averaged, to arrive at a single index of declarative memory performance. This index was scaled (see below) and was treated as our measure of declarative memory in our primary analyses.

In order to assess the reliability and internal consistency of this performance measure, we used the package “psych” (Revelle and Revelle, 2015) in R (R Development Core Team, 2017; Version 3.4.1, 2017) to calculate Cronbach’s alpha (α) and Guttman’s lambda 6 (λ_6_). The scaled declarative memory scores had α = 0.59 and λ*_6_* = 0.75 on Day 1, and α = 0.60, and λ*_6_* = 0.76 on Day 2. These values for α are considered to be in the “low” to “acceptable” range, and values for λ_6_ are considered to be in the “good” range (Guttman, 1945; Cronbach, 1951).

A two-tailed, paired samples *t*-test conducted on day 1 vs. day 2 declarative memory scores suggests that on average, participants increased their recognition memory accuracy after the overnight delay, *t*(78) = -4.65, *p* < 0.001, 95% CI [-0.079, -0.032], *Cohen’s d* = 0.57. This observation is consistent with prior accounts of enhanced declarative memory retrieval following a period of sleep (Diekelmann and Born, 2010).

#### Procedural Memory

For the serial reaction time task, trial-by-trial accuracy data was inspected to ensure that all participants understood and were engaged with the task (all participants achieved >90% accuracy over all blocks). Prior to calculating the average reaction time per person per block, reaction time data from incorrect trials were removed. Procedural memory performance was defined as the difference in reaction time between the last random block and the last sequence block for each of the two respective sessions (Lum et al., 2010). This difference score was scaled (see below) and was treated as our measure of procedural memory in our primary analyses below.

We followed the same procedures as described above to assess the reliability and internal consistency of this measure. The scaled procedural memory scores had α = 0.61 and λ*_6_* = 0.67 on Day 1, and α = 0.62, and λ_6_ = 0.72 on Day 2. These values are all considered with the “acceptable” range of reliability and internal consistency (Guttman, 1945; Cronbach, 1951).

In order to determine if performance differed across the two sessions, we conducted a two-tailed, paired samples *t*-test conducted on day 1 vs. day 2 procedural memory scores. This comparison suggested that procedural memory performance declined across days, *t*(78) = -3.85, *p* < 0.001, 95% CI [0.035, 0.109], *Cohen’s d* = 0.43. This observation is consistent with previous reports that sleep may not necessary benefit implicitly learned motor sequences (Nemeth et al., 2010).

#### Correlations Between Experimental Measures

In order to examine the extent to which task performances were mutually associated, we conducted an initial correlational analysis on the scaled scores across experimental tasks. After applying the Holms-Bonferroni correction to account for family-wise error rate, we found that Day 1 procedural memory performance was significantly correlated with Day 2 procedural memory performance, and that Day 1 declarative memory performance was significantly correlated with Day 2 declarative memory performance (see Table 2). Procedural and Declarative memory performances were not significantly correlated on either day. These patterns demonstrate two important points about our tasks. First, that performance on the procedural memory tasks appear to be relatively independent of declarative memory performance (and vice versa). Second, the within-task associations across days illustrate relative within-group stability in performance across days, despite group-level changes observed (above) on declarative memory performance.

**TABLE 2:** Correlation matrix of scaled performance on experimental tasks across days.

	PM Day 1	PM Day 2	DM Day 1
**PM Day 2**	*R* = 0.29, *p* = 0.001*		
**DM Day 1**	*R* = 0.16, *p* = 0.156	*R* = 0.12, *p* = 0.309	
**DM Day 2**	*R* = 0.09, *p* = 0.409	*R* = 0.11, *p* = 0.347	*R* = 0.62, *p* < 0.001*

Table displays correlations across scaled performances on experimental tasks across days. *Indicates statistical significance at.05 level following Holms-Bonferroni adjustment of *p*-values to account for family-wise error rate.

### Data Transformations

In order to ensure that our measures of declarative and procedural memory are on commensurate scales, the outcome variables for the serial reaction time task and the object recognition memory tasks were transformed according to the proximity-to-maximum scaling method (Moeller, 2015). This method was chosen over Z-standardization because it preserves both the between-subjects variability within each time point as well as the within-subjects variability over repeated measures.

In addition, we applied the same transformation to the raw scores obtained on the phonological processing assessments, because a different version of the CTOPP was administered at each site. Furthermore, as Elision and Blending are both considered measures of PA, and because performance on these two measures were highly collinear (*R* = 0.92), scaled scores from these two subtests were averaged into a single index of PA.

### Relationships Between Phonological Processing and Memory Performance

#### Phonological Awareness

In order to examine the relationship between PA and memory abilities assessed shortly after learning, we first conducted a multiple regression on PA with the Session 1 declarative and procedural memory scores as the two predictors, with site entered as a covariate. This model did not significantly account for individual differences in PA, *F*(3,75) = 2.11, *r^2^ adj* = 0.04, *p* = 0.107 (see Table 3A for parameter estimates).

**TABLE 3:** Results for multiple regression analysis of phonological awareness (PA) scores.

(A) Day 1 memory scores as predictors				

	*ß*	*SE ß*	*t*	*p*
Intercept	0.78	0.05	16.68	<0.001***
Declarative memory	0.10	0.07	1.49	0.140
Procedural memory	0.07	0.04	1.53	0.129
Site	0.00	0.01	−0.11	0.910

**(B) Day 2 memory scores as predictors**				

	***ß***	***SE ß***	***t***	***p***

Intercept	0.78	0.05	15.90	<0.001***
Declarative memory	0.15	0.06	2.45	0.016*
Procedural memory	0.00	0.06	−0.01	0.996
Site	0.01	0.02	0.50	0.622

*Denotes statistical significance at 0.05 level. ***Denotes statistical significance at 0.001 level.

**FIGURE 1:**
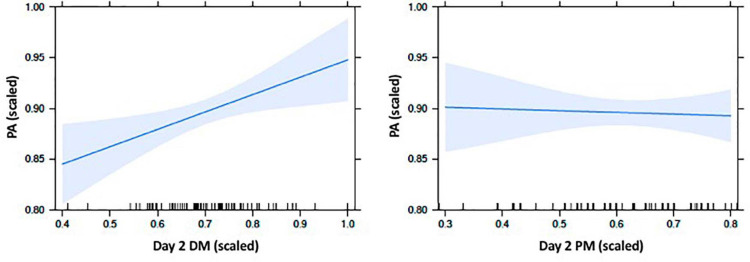
Effect plots for Session 2 memory abilities on phonological awareness (PA). Figure depicts the relative predictive relationships between PA and declarative memory (DM) and PA and procedural memory (PM), assessed after an overnight delay, and controlled for site. Values for all variables are scaled according to the proximity-to-maximum method (Moeller, 2015). Graphs were generated using the package “effects” in R (Fox et al., 2016). Shaded regions represent the pointwise confidence band for the predictor effects, based on standard errors calculated from the covariance matrix of the fitted regression coefficients (Fox and Weisberg, 2018).

We then examined if there may be a delayed emergence between PA and memory abilities when assessed after an overnight delay (as observed for acoustic-phonetic memory in Earle and Arthur, 2017). We regressed PA with the Session 2 declarative and procedural memory scores, with site as covariate. This model was marginally significant, *F*(3,75) = 2.54, *r^2^ adj* = 0.06, *p* = 0.06, driven by a significant association with declarative memory, but not procedural memory, after controlling for site (see Table 3B for parameter estimates). Figure 1 depicts relationships between PA and memory abilities at Session 2, each adjusted for the other predictors in the model.

#### Non-word Repetition

**FIGURE 2:**
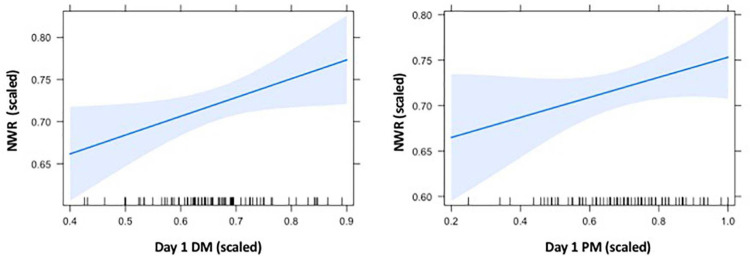
Effect plots for Session 1 memory abilities on nonword repetition (NWR). Figure depicts the relative predictive relationships between NWR and DM assessed shortly after learning, and NWR and PM, and controlled for site. Values for all variables are scaled according to the proximity-to-maximum method (Moeller, 2015). As in Figure 1, graphs were generated using the package “effects” in R (Fox et al., 2016), and shaded regions represent the pointwise confidence band for the predictor effects.

In order to examine the relationship between NWR and memory abilities assessed shortly after learning, we first regressed PA with the Session 1 declarative and procedural memory scores as the two predictors, with site entered as a covariate. This model significantly accounted for individual differences in NWR scores, *F*(3,75) = 18.27, *r^2^ adj* = 0.40, *p* < 0.001. This model included a significant association with declarative memory, as well as site, but not procedural memory (see Table 4A for parameter estimates). Figure 2 depicts relationships between NWR and memory abilities at Session 1, each adjusted for the other predictors in the model.

**TABLE 4:** Results for multiple regression analysis of nonword repetition (NWR) scores.

(A) Day 1 memory scores as predictors				

	*ß*	*SE ß*	*T*	*p*
Intercept	0.57	0.07	7.85	<0.001***
Declarative memory	0.22	0.10	2.19	0.032*
Procedural memory	0.11	0.07	1.62	0.110
Site	−0.13	0.02	−5.95	<0.001***

**(B) Day 2 memory scores as predictors**				

	*ß*	*SE ß*	*T*	*p*

Intercept	0.66	0.08	8.31	<0.001***
Declarative memory	0.09	0.10	0.88	0.382
Procedural memory	0.13	0.10	1.20	0.233
Site	−0.14	0.03	−5.45	<0.001***

*Denotes statistical significance at 0.05 level. ***Denotes statistical significance at 0.001 level.

We then examined if there may be a delayed emergence between NWR and memory abilities when assessed after an overnight delay. We regressed NWR with the Session 2 declarative and procedural memory scores, with site as covariate. This model significantly accounted for individual differences in NWR, *F*(3,75) = 13.92, *r^2^ adj* = 0.33, *p* < 0.001, however, this was driven by site, and not by neither of our two predictors of interest (see Table 4B for parameter estimates).

In summary, we found that declarative memory performance was predictive of phonological processing in adults. Specifically, declarative memory assessed after a 12-h delay was associated with PA. Declarative memory assessed shortly after learning was associated with NWR. Experiment site, and by extension, the test version that was administered, was also associated with NWR. We discuss the possible implications of these findings below.

## Discussion

This study explored contributions of two learning and memory systems to performance on phonological processing tasks in adults with typical language and reading abilities. Specifically, we investigated the extent to which measures of declarative and procedural memory related to performance on PA and NWR tasks. Below, we discuss each finding within the context of the previous literature.

First, we found that PA was associated with declarative memory when it was assessed after a 12-h delay, but not when tested shortly after learning. This finding resonates with a previous relationship between PA and speech-perceptual memory that similarly emerged only after an overnight delay (Earle and Arthur, 2017). Taken together with the current findings, this may mean that PA performance in adults depends, at least in part, on access to information after it has been learned and consolidated into its long-term state in declarative memory. In the context of PA performance, the metalinguistic demands of the task (e.g., phonological working memory, auditory attention) are likely influenced by one’s ability to retrieve long-term speech-sound representations (Pierrehumbert, 2016). Indeed, Earle and Arthur (2017) speculated that this critical long-term memory was of the speech sound representations. The current study may suggest that this relationship with memory is not domain-specific to speech, but to declarative memory more broadly. Alternatively, the association between PA and delayed declarative memory may reflect a relationship between task performance on PA and long-term lexical knowledge, which is thought to be learned primarily in declarative memory (Ullman, 2004; Ullman et al., 2020). For example, the Blending task involves matching a sequence of individuated phonological segments onto a lexical item, and thus the quality of lexical representations may determine how readily one is able to complete this task.

Second, we found that NWR was associated with declarative memory when it was assessed shortly after learning, but not after a 12-h delay. As discussed previously, NWR performance relies at least partly on lexical knowledge (Edwards et al., 2004). Thus, we expected at least some relationship between declarative memory and NWR performance. This expectation was tempered by the use of the CTOPP-2 NWR task, the stimuli for which are not as word-like as in some other NWR tasks (e.g., Children’s Test of NWR, Gathercole and Baddeley, 1996; see Arthur, 2017, on NWR task comparisons). Furthermore, we cannot take for granted that NWR ability in adulthood reflects differences in lexical inventory as it does in childhood. Also, we had expected this relationship to emerge after a delay (similar to the association with PA above, and the relationship observed with speech learning in Earle and Arthur, 2017), rather than shortly after learning. This may mean that NWR performance relies on similar mechanisms as in the recognition memory task when tested shortly after learning. For example, familiarity of stored information may play a role in both tasks, in that familiarity with common sublexical phonological strings is linked to better performance in NWR (Gupta and Tisdale, 2009), while familiarity with objects in the encoding phase of recognition memory tasks is linked to better memory for these objects minutes later during the recognition phase (Reifegerste et al., 2021). Alternatively, the association observed here between NWR and declarative memory may point to the involvement of the hippocampus during working memory tasks (Yonelinas, 2013), or the involvement of working memory and attentional resources common to both processing and memory formation tasks (Chun and Turk-Browne, 2007). Further investigation is necessary to determine to which of these possibilities are likely to best explain the association.

Nonword repetition performance was also associated with experiment site, which was entered as a covariate, and by extension, the test version that was administered. As a reminder, we used a test version that had a lower ceiling at the first site (UConn). We suspect that it is for this reason that we found differences in performance across sites, even after scaling. We do not believe this to have had an impact on our other findings, because we statistically controlled for site in each of our regression models. Nonetheless, this is a potential limitation.

Our findings collectively suggest that declarative memory, and not procedural memory, is associated with phonological processing skills in adulthood. Given the similarity between the slopes in the regression lines of declarative and procedural memory on NWR performance (Figure 2), there may yet be subtle influences of procedural memory on NWR (e.g., the lack of statistical significance may be due to larger variance of scores within procedural vs. within declarative memory). This would be consistent with the argument that procedural memory is involved in the concatenation of phonological segments (as suggested in Dye et al., 2016) as well as in the building of speech sound representations (Ullman et al., 2020). However, it may also be the case that procedural memory is unrelated to phonological processing ability in adulthood. This may, in part, be attributable to our examination of individuals with intact mature language capabilities. As mentioned in the introduction, phonotactic rules may have been largely learned in procedural memory during childhood, and thus procedural learning abilities during adulthood may be less relevant. If this is the case, individual differences in phonological processing in adulthood may be driven instead by differences in lexical and phonological representational quality. If this study were to be carried out in a developing population, one might expect that performance on phonological processing tasks would be more reliant on information learned by procedural memory.

In more general terms, the current findings offer empirical support for the idea that individual differences in phonological processing may be attributable to individual differences in learning and memory abilities. This may have implications for how we consider weaknesses in phonological processing in different populations. There may be potential practical applications, such as leveraging declarative memory to support phonological processing performance in adults.

There are some important limitations to consider in the interpretations of our findings above. First, we reiterate that this dataset examines an adult sample, and thus the relationships between memory and phonological processing abilities may be different during development. Second, the adult samples were selected from amongst university populations, and thus performance may reflect those at the higher end of the distribution, limiting the generalizability of our findings. Third, our interpretation of memory as assessed immediately vs. following a delay may be tempered by the scheduling of our tasks as taking place in the evening (Day 1) vs. morning (Day 2). Thus, the relationships between declarative memory and phonological processing may have been partially attributable to circadian effects. This concern is somewhat mitigated by the fact that testing of phonological processing occurred at different times of day. Finally, this study places a heavy burden on the interpretation of task performance to measure individual traits (such as memory and phonological processing skill). The current findings may be strengthened by replicating these results through estimating these traits through multiple measures in the future.

Despite the above limitations, the current study offers some interesting directions for the future. Follow-up investigations of phonological processing and memory skills may consider prospectively testing the predictive relationships between declarative and procedural memory in childhood and the development of phonological processing, both in typical and impaired populations. Further, it may be revealing to track changes over time to these relationships longitudinally. Interestingly, a similar pattern has been recently observed in the association between language skills and declarative, but not procedural, memory in adults (Llompart and Da̧browska, 2020). Thus, the current findings may point to a broader narrative concerning the robust relationship between language and declarative memory in adulthood.

In conclusion, the current work provides an interesting addition to the ongoing discussion of individual differences in phonological processing tasks. Specifically, this study begins to explore the relationships between aspects of phonological processing and declarative and procedural memory tasks. Relationships between both PA and NWR and declarative memory were revealed. To our knowledge, this is the first empirical test of whether phonological processing abilities relate to individual differences in domain-general learning and memory abilities. The findings invite questions and further investigations regarding the nature of the relationships between phonological processing and memory systems across different populations.

## Data Availability Statement

Publicly available datasets were analyzed in this study. This data can be found here: https://osf.io/gbdxs/?view_only=274f09f334fc4f0cb221158cd70a2974.

## Ethics Statement

The studies involving human participants were reviewed and approved by University of Connecticut Institutional Review Board and University of Delaware Institutional Review Board. The patients/participants provided their written informed consent to participate in this study.

## Author Contributions

The current manuscript was written in collaboration between DA and FE. Data analysis was performed by FE. MU contributed theoretical framing and interpretation of the work. All three authors were heavily involved in finalizing the draft. All authors contributed to the article and approved the submitted version.

## Conflict of Interest

The authors declare that the research was conducted in the absence of any commercial or financial relationships that could be construed as a potential conflict of interest.
